# Antimicrobial Activity of Natural Extracts Against Catheter-Colonizing Methicillin-Resistant *Staphylococcus aureus* Clinical Isolates

**DOI:** 10.3390/biomedicines13092150

**Published:** 2025-09-04

**Authors:** José Avendaño-Ortiz, Alba Tribaldo, Luna Ballestero, Luis Antonio Gómez, Ignacio Gracia, Juan Francisco Rodríguez, Natalia Bejarano Ramírez, Raquel Bodoque-Villar, María Ángeles Vaz-Salgado, Rosa del Campo, Francisco Javier Redondo-Calvo

**Affiliations:** 1 Department of Microbiology, Hospital Universitario Ramón y Cajal and Instituto Ramón y Cajal de Investigación Sanitaria (IRYCIS), 28034 Madrid, Spain; joseavenort@gmail.com (J.A.-O.); alba.tribaldo@hotmail.com (A.T.); luna.ballestero@gmail.com (L.B.); 2 CIBERINFEC, Instituto de Salud Carlos III, 28029 Madrid, Spain; 3 Translational Research Group, GAI of Ciudad Real, Research Institute of Castilla-La Mancha (IDISCAM), 13004 Ciudad Real, Spain; luisantonio.gomez@alvenpesalud.com (L.A.G.); ignacio.gracia@uclm.es (I.G.); juan.rromero@uclm.es (J.F.R.); nbejarano@sescam.jccm.es (N.B.R.); rbodoquev@sescam.jccm.es (R.B.-V.); 4 Department of Chemical Engineering, Institute of Chemical and Environmental Technology, Universidad de Castilla-La Mancha, 13071 Ciudad Real, Spain; 5 Faculty of Medicine, Universidad de Castilla-La Mancha, 13071 Ciudad Real, Spain; 6 Department of Pediatrics, University General Hospital, 13004 Ciudad Real, Spain; 7 Translational Research Unit, University General Hospital and Research Institute of Castilla-La Mancha (IDISCAM), 13004 Ciudad Real, Spain; 8 Medical Oncology Department, Hospital Universitario Ramón y Cajal and Instituto Ramón y Cajal de Investigación Sanitaria (IRYCIS), 28034 Madrid, Spain; 9 Department of Anesthesiology and Critical Care Medicine, University General Hospital, 13004 Ciudad Real, Spain

**Keywords:** *Staphylococcus aureus* (*S. aureus*), methicillin-resistant *Staphylococcus aureus* (MRSA), chlorhexidine gluconate (CHG), natural extract, garlic, grape, propolis, antimicrobial, Minimum Inhibitory Concentration (MIC), Minimum Bactericidal Concentration (MBC)

## Abstract

**Background:** Intravascular catheters (ICs) are critical medical devices but require frequent replacement due to the risk of bacterial colonization, which can lead to bloodstream infections. This process causes patient discomfort and incurs significant health and economic costs. **Aim:** To evaluate the inhibitory activity of natural extracts as potential IC coatings to prevent colonization by methicillin-resistant *Staphylococcus aureus* (MRSA). **Methods:** Thirty-six clinical MRSA isolates, obtained from ICs using the Maki technique, were tested. Three natural extracts were evaluated: garlic extract enriched in thiosulfinates (allicin: 7 mg/g), grape extract enriched in proanthocyanidins (92% proanthocyanidins), and propolis extract. Chlorhexidine gluconate (CHG) served as the bactericidal control. The minimum inhibitory concentration (MIC) was determined using the broth microdilution technique with optical density measurements and resazurin-based viability confirmation. The minimum bactericidal concentration (MBC) was assessed from viable cells in wells exceeding the MIC. **Results:** All tested extracts exhibited bacteriostatic activity against MRSA isolates. The grape extract demonstrated the lowest MIC_90_ (3.125 mg/mL), followed by propolis extract (MIC_90_ = 12.5 mg/mL) and garlic extract (MIC_90_ = 50 mg/mL). Only the propolis extract showed bactericidal activity (MBC = 25 mg/mL). While CHG outperformed the natural extracts, their activity against MRSA suggests potential clinical utility. **Conclusion:** The natural extracts studied display promising bacteriostatic activity against MRSA isolates from ICs, with propolis extract additionally showing bactericidal effects. Although less potent than CHG, these extracts offer a potential alternative for combating multidrug-resistant pathogens in clinical settings, warranting further investigation for use as IC coatings.

## 1. Introduction

*Staphylococcus aureus* is a common commensal microorganism but also a leading cause of severe infections and mortality worldwide, particularly due to methicillin-resistant *S. aureus* (MRSA) strains [1]. Healthcare workers are significant carriers, with 11–45% colonized by *S. aureus* and 1–25% carrying MRSA often on work clothing [2,3]. In healthcare settings, Intensive Care Units (ICUs) are relevant MRSA reservoirs, with up to 12% of hospitalized patients in the UK encountering MRSA during admission and an additional 12% acquiring it in ICUs [1].

Current MRSA decolonization relies on chlorhexidine gluconate (CHG), a topical biocide that disrupts bacterial cell walls and membranes, typically applied through wipes or daily baths, often combined with mupirocin [4,5]. Beyond patient decolonization, environmental decontamination is critical to prevent nosocomial infections. *S. aureus* adheres persistently to hospital surfaces, with intravascular catheters (ICs) serving as primary entry points for environmental microorganisms. Frequent catheter replacement is required to prevent microbial adhesion and biofilm formation by skin microbiota, such as *S. aureus* [1].

Rising antibiotic resistance necessitates novel antimicrobial strategies. Polyphenol-rich natural extracts from propolis, grape, and garlic show promise due to their reported anti-inflammatory, antiseptic, and immunomodulatory properties [6,7,8,9,10,11,12,13]. These extracts exhibit direct antimicrobial activity or enhance conventional antibiotics, potentially reducing resistance development [9,10,14]. Emerging products leveraging these extracts, particularly for catheter coatings or environmental decontamination, utilize their food-grade status to minimize co-selection of antibiotic- and disinfectant-resistant strains [15,16].

This study evaluates the antimicrobial activity of three food-grade natural extracts—garlic (enriched in thiosulfinates), grape (enriched in proanthocyanidins), and propolis—against MRSA clinical isolates from ICU patients’ ICs, compared to CHG. While prior studies have reported the antibacterial properties of these extracts, this research uniquely assesses polyphenol-enriched formulations against a clinically relevant MRSA collection from colonized ICs, determining their bacteriostatic or bactericidal potential.

## 2. Materials and Methods

### 2.1. Bacterial Clinical Isolates

A collection of 36 MRSA clinical isolates was obtained from the tips of ICs from independent patients admitted to the ICU of Ramón y Cajal University Hospital (Madrid, Spain) over a 10-year period. The isolates were recovered using the Maki semi-quantitative technique, which involves rolling the catheter tip across an agar plate to assess microbial colonization. Following initial isolation, the isolates were routinely frozen for storage. For this study, the isolates were re-identified using matrix-assisted laser desorption/ionization time-of-flight mass spectrometry (MALDI-TOF; Bruker Daltonik MALDI Biotyper^®^, Bremen, Germany) to confirm their identity as *S. aureus*. Methicillin resistance was verified through cefoxitin susceptibility testing performed on a MicroScan WalkAway system (Beckman Coulter, CA, USA). The isolates were stored at −20 °C until required for experimental use. Prior to experiments, the isolates were thawed and subcultured on Brain Heart Infusion (BHI) agar (Difco, NJ, USA) and incubated for 24 h at 37 °C to ensure viability and purity.

### 2.2. Food-Grade Natural Extracts

Three natural extracts were evaluated for their antimicrobial properties: garlic extract (*Allium sativum*, EAST), grape extract (*Vitis vinifera*, EVVP), and propolis extract (EP). The garlic extract was derived from purple garlic sourced from Las Pedroñeras (Cuenca, Spain). It was prepared through lyophilization and enriched with thiosulfinates, yielding a final allicin concentration of 7.03 mg/g. The grape extract was obtained from *Vitis vinifera* via lyophilization and a proanthocyanidin enrichment process, achieving a final proanthocyanidin concentration of 92%. The detailed composition of both extracts was determined using High-Performance Liquid Chromatography (HPLC) and are presented in Table 1 and Table 2, respectively. Table 1 lists the absolute concentrations of identified bioactive compounds in garlic extract, while Table 2 outlines the composition and relative percentages of major phenolic compounds in grape extract. The production processes for both extracts are protected by patents (see Patent section for details). The propolis extract was supplied by González Carballal (Ourense, Spain) as a tincture comprising 30% propolis and 70% brandy (40% alcohol by volume).

### 2.3. Antimicrobial Susceptibility to Natural Extracts

The minimum inhibitory concentration (MIC) was determined using the broth microdilution technique in 96-well plates, following the European Committee on Antimicrobial Susceptibility Testing (EUCAST) guidelines (https://www.eucast.org/clinical_breakpoints, accessed on 21 July 2025). Assays were conducted in Müller–Hinton broth (Difco, NJ, USA) with a bacterial inoculum of approximately 5 × 10^5^ CFU/mL, prepared by diluting a 0.5 McFarland standard. For garlic and grape extracts, 1 g of lyophilized extract was dissolved in 10 mL of sterile saline to create stock solutions of 100 mg/mL, which were sterilized using a 0.8-micron filter. Two-fold serial dilutions in Müller–Hinton broth were performed in 96-well plates, yielding final concentrations ranging from 0.1875 mg/mL to 50 mg/mL for both extracts. The propolis extract, a commercial 30% *w*/*v* (300 mg/mL) liquid extract, was filtered and serially diluted in Müller–Hinton broth to achieve concentrations from 0.78 mg/mL to 50 mg/mL. The 40% alcohol content of the stock was accounted for by including negative controls with the alcohol vehicle diluted in Müller–Hinton broth in equivalent proportions. CHG, a 20% *w*/*v* (200 mg/mL) aqueous solution (Acofarma, Madrid, Spain), served as the bactericidal positive control, tested at concentrations from 0.01 µg/mL to 2 mg/mL.

After 24 h of incubation at 37 °C, bacterial growth was quantified by measuring optical density at 600 nm (OD_600_) using a microplate reader (Tecan Infinite 200 PRO). Additionally, 10 µL of 1.3 µM resazurin (Sigma-Aldrich, St. Louis, MO, USA) was added to each well and incubated for 2 h to assess metabolic activity and cell viability. The MIC was defined as the lowest concentration preventing visible growth, indicated by the blue/purple color of resazurin. All experiments were performed in triplicate, including positive and negative controls. To account for the intrinsic color of the extracts, control wells containing sterile broth and each extract concentration (without inoculum) were included for background subtraction. MIC values were confirmed visually and via the resazurin viability assay. The reference strain *S. aureus* ATCC 29213 was included in each assay run for quality control using the EUCAST guidelines.

To determine the minimum bactericidal concentration (MBC), 10 µL from wells corresponding to the MIC and higher concentrations were plated onto Columbia blood agar plates (Thermo Scientific, Waltham, MA, USA) and incubated for 24 h at 37 °C. The MBC is defined as the lowest concentration that results in no visible bacterial growth on the agar plates.

### 2.4. Statistical Analysis

To investigate potential relationships between the antimicrobial activities of the natural extracts, pairwise correlation analysis was performed using the MIC values obtained for 36 MRSA clinical isolates. Pearson’s correlation coefficient (r) was calculated to quantify the linear relationship between the MIC values for each pair of extracts. A *p*-value less than 0.05 was considered indicative of a statistically significant correlation. The analysis was conducted using the SciPy library in Python (version 3.9). Results were visualized as a scatter plot matrix generated with the Seaborn library (version 0.12), facilitating the identification of patterns and correlations in the antimicrobial activities of the extracts.

## 3. Results

### 3.1. Bacteriostactic Effects of the Natural Extracts

**Figure 1:**
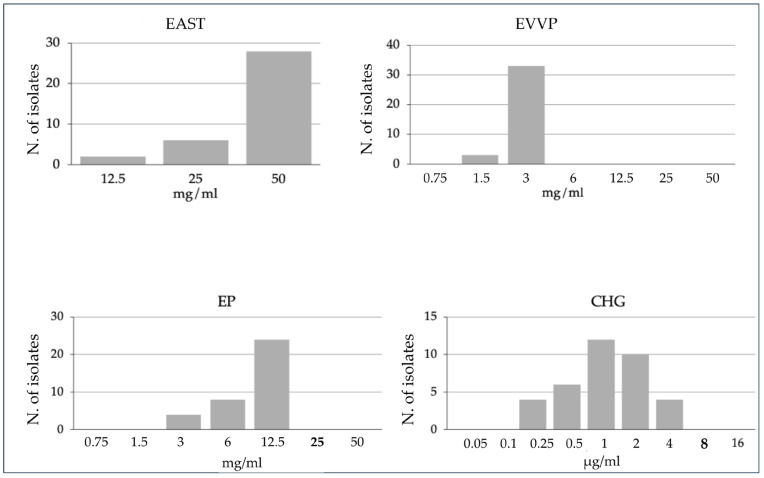
Frequency distribution histograms of minimum inhibitory concentration (MIC) values for chlorhexidine (CHG), garlic extract (EAST), grape extract (EVVP), and propolis extract (EP) against 36 MRSA isolates from intravascular catheters of ICU-admitted patients. The *y*-axis represents the number of isolates inhibited at a given concentration. The *x*-axis shows the serial dilutions of the compounds. The minimum bactericidal concentration (MBC), where applicable, is indicated in bold on the *x*-axis. For detailed MIC and MBC results for each individual isolate, please see Supplementary Table S1.

The three food-derived extracts demonstrated bacteriostatic activity against the MRSA collection, and detailed MIC results for each isolate are provided in Supplementary Table S1. The garlic extract required the highest concentrations for inhibition, with both MIC_50_ and MIC_90_ values of 50 mg/mL. In contrast, the grape extract exhibited the highest inhibitory activity, with MIC_50_ and MIC_90_ values of 3 mg/mL. The propolis extract showed intermediate inhibitory activity, with MIC_50_ and MIC_90_ values of 12.5 mg/mL. The distribution of MIC values across the isolates was generally consistent, clustering within two to three dilution steps for each extract, as illustrated in Figure 1.

All isolates remained susceptible to CHG, with four isolates exhibiting an MIC of 4 µg/mL, which aligns with the previously established susceptibility breakpoint. The MIC distribution for CHG was also homogeneous, clustering within two to three dilution steps, as shown in Figure 1.

### 3.2. Bactericidal Activity of the Natural Extracts

Among the tested natural extracts, only the propolis extract demonstrated bactericidal activity, with a consistent MBC of 25 mg/mL across all isolates. In contrast, neither the garlic nor the grape extracts exhibited bactericidal effects at the highest concentrations tested (up to 50 mg/mL). CHG, used as the bactericidal control, showed bactericidal activity at concentrations ≥ 4 µg/mL for all isolates.

### 3.3. MICs of the Natural Extracts Do Not Exhibit Positive Correlations Between Them

To explore potential relationships in the susceptibility of our clinical isolates to the tested natural extracts, a pairwise correlation analysis of the MICs was conducted. The results are visualized in a scatter plot matrix (Supplementary Figure S1). No significant positive correlations were observed between the antimicrobial activities of the extracts, suggesting a lack of cross-susceptibility among them. This may be influenced by the high homogeneity in MIC values across isolates, as previously noted.

Notably, a weak but statistically significant negative correlation was identified between the MICs of garlic and propolis extracts (Pearson’s r = −0.36, *p* = 0.032; Supplementary Figure S1). This indicates that isolates more resistant to garlic were slightly more susceptible to propolis. No statistically significant correlations were found between the MICs of garlic and grape extracts (r = −0.16, *p* = 0.359) or between grape and propolis extracts (r = −0.03, *p* = 0.864). These findings suggest that the bacteriostatic mechanisms of the three extracts are likely to differ, contributing to their distinct antimicrobial profiles.

## 4. Discussion

The three food-derived extracts demonstrated bacteriostatic activity against MRSA clinical isolates from ICs, consistent with their expected efficacy against Gram-positive bacteria due to their permeable cell membranes [17]. The grape extract exhibited the highest bacteriostatic activity (MIC_90_ = 3.125 mg/mL), followed by propolis (MIC_90_ = 12.5 mg/mL) and garlic (MIC_90_ = 50 mg/mL). Notably, only propolis displayed bactericidal activity, with a consistent MBC of 25 mg/mL across all 36 isolates, highlighting its potential for applications requiring bacterial killing. In contrast, garlic and grape extracts lacked bactericidal effects at the tested concentrations, while CHG was bactericidal at ≥4 µg/mL, reflecting its established clinical efficacy [3,4].

Garlic’s antimicrobial activity, primarily driven by allicin (70–80% of thiosulfinates), disrupts bacterial redox balance by reacting with thiol-group-containing enzymes [18,19,20,21]. However, its high MIC_90_ (50 mg/mL) and lack of bactericidal activity suggest limited potency compared to modern biocides [19]. Grape extract’s proanthocyanidins, comprising up to 70% of phenolic compounds, alter bacterial membrane permeability and may produce toxic oxidative molecules [22,23,24,25]. Its low MIC_90_ indicates strong bacteriostatic potential, potentially enhanced by synergistic effects with antibiotics [26]. Propolis, with its complex composition of polyphenols, waxes, and aromatic acids, disrupts bacterial membrane potential, ATP production, and motility, offering both bacteriostatic and bactericidal effects [27]. The bactericidal activity of propolis extract was attributed to propolis, not its ethanol vehicle, as control dilutions showed no antimicrobial effects.

All isolates were susceptible to CHG, with MICs ≤ 4 µg/mL, consistent with clinical concentrations (1–2%) that minimize resistance selection [3]. While *qac*A/B genes may confer CHG resistance in MRSA, no significant resistance was observed, and methicillin resistance did not affect CHG susceptibility [4]. The lack of positive correlation between MICs of the extracts (r = −0.03 to −0.36) suggests distinct antimicrobial mechanisms, reducing the risk of cross-resistance and supporting their potential use in combination or rotation strategies.

Limitations include potential interference from the extracts’ chromogenic properties at high concentrations and the lack of detailed compositional data for the commercial propolis tincture. While appropriate controls were included, excipient effects and bacterial growth variability could not be fully excluded. The absence of an antimicrobial neutralization step in MBC testing may have influenced results, though this was likely negligible for garlic and grape extracts due to their lack of bactericidal activity. Additionally, MIC and MBC values do not fully replicate the complex environment of catheter-associated biofilms, necessitating further studies with in vitro biofilm models and coated catheter segments to assess stability, release kinetics, and anti-biofilm efficacy.

The food-grade status of these extracts offers a safety advantage, enabling high local concentrations on catheter surfaces with minimal systemic exposure. This could allow their use at levels toxic to synthetic biocides, providing a strategy against multidrug-resistant organisms without contributing to resistance selection. Future research should focus on toxicity assays and preclinical models to validate the efficacy and safety of these extracts as catheter coatings.

## 5. Conclusions

In summary, propolis extract demonstrated significant bacteriostatic and bactericidal activity against MRSA isolates from ICs of ICU patients. In contrast, garlic and grape extracts exhibited bacteriostatic activity but lacked bactericidal effects at the tested concentrations. CHG outperformed all extracts, achieving both inhibition and bacterial killing at low concentrations (≥4 µg/mL). Despite CHG’s superior efficacy, the food-grade natural extracts, particularly propolis, offer a promising alternative for preventing MRSA colonization and biofilm formation on ICs. Their use as catheter coatings could reduce the risk of biocide-resistant isolates, leveraging their safety profile to maintain high local concentrations without systemic toxicity. Further studies are warranted to validate their efficacy in biofilm models and clinical settings.

## 6. Patents

Patent WO 2008/102036 A1. Method for obtaining a freeze-dried, stable extract from plants of the *Allium* genus.

National patent (Spanish Trademark number ES2675282A1). *Allium sativum* extract, its use for the manufacture of a medicinal product for the treatment of diseases, and its obtaining procedure. Spanish national patent application number 0000028810 for the manufacture and obtaining procedure of a nutraceutical polyphenol-enriched product from *Vitis vinifera* and other plants.

## Figures and Tables

**Table 1:** Garlic extract (EAST) composition.

Compound	Concentration (mg/kg)
Total polyphenols	13,910
Total flavonoids	3220
Diallyl thiosulfinates (allicin)	7030
S-allyl-L-cysteine	80
Leucine	586
Isoleucine	500
Valine	477
Methionine	316
Cysteine	811
Phenylalanine	556
Tyrosine	4499
Aspartic acid	901
Glutamic acid	2866
Arginine	4090
Lysine	617
Histidine	891
Threonine	812
Serine	385
Glycine	215
Alanine	897
Thiamine (B1)	552
Riboflavin (B2)	2
Niacin (B3)	26
Pantothenic acid (B5)	1556
Biotin (B7)	251
Cobalamin (B12)	898
Ascorbic acid (C)	3347
Linoleic acid (F)	276
Tocopherol (E)	7
Menadione (K3)	7

**Table 2:** Grape extract (EVVP) composition.

Compound	Concentration (mg/kg)	Concentration (%)
Procyanidin B1	58.32	1.17
Catechin	105.77	2.12
Procyanidin B2	48.15	0.96
Epicatechin	117.74	2.35
Procyanidin C1	30.52	0.61
Proanthocyanidins	4624.18	92.48
Total flavonoids and polyphenols	4984.67	99.78

## Data Availability

The raw data supporting the conclusions of this article will be made available by the authors on reasonable request.

## Supplementary Materials

The following supporting information can be downloaded at https://www.mdpi.com/article/10.3390/biomedicines13092150/s1, Figure S1: Pairwise correlation analysis of Minimum Inhibitory Concentrations (MICs) for the three natural extracts; Table S1: Minimum Inhibitory Concentration (MIC) and Minimum Bactericidal Concentration (MBC) of garlic extract (EAST), grape extract (EVVP), and propolis extract (EP) against 36 clinical MRSA isolates.

## Abbreviations

The following abbreviations are used in this manuscript:

CHG	Chlorhexidine Gluconate
EAST	Extract of *Allium sativum* enriched in Thiosulfinates
EP	Propolis Extract
EVVP	Extract of *Vitis vinifera* enriched in Proanthocyanidins
IC	Intravascular Catheter
ICU	Intensive Care Unit
MBC	Minimum Bactericidal Concentration
MIC	Minimum Inhibitory Concentration
MRSA	Methicillin-Resistant *Staphylococcus aureus*
