# Supplementary material for: Antimicrobial Activity of Natural Extracts Against Catheter-Colonizing Methicillin-Resistant Staphylococcus aureus Clinical Isolates
Source: Biomedicines. 2025 Sep 4;13(9):2150. doi: 10.3390/biomedicines13092150 (PMC12467437; doi:10.3390/biomedicines13092150)
Supplement: Supplementary file 1 [file biomedicines-13-02150-s001.zip › biomedicines-3822912-SI.pdf]

SUPPLEMENTARY MATERIAL

Supplementary Table S1: Minimum Inhibitory Concentration (MIC) and Minimum Bactericidal Concentration (MBC) of garlic extract (EAST), grape extract (EVVP), and propolis extract (EP) against 36 clinical MRSA isolates.

	EAST (mg/mL)		EVVP (mg/mL)		EP (mg/mL)	
**Isolate**	**MIC**	**MBC**	**MIC**	**MBC**	**MIC**	**MBC**
1	50.0	>50	3.124	>100	6	25
7	25.0	>50	3.124	>100	12.5	25
8	50.0	>50	3.124	>100	12.5	25
10	50.0	>50	3.124	>100	6	25
11 (2003)	12.5	>50	3.124	>100	12.5	25
11 (2005)	50.0	>50	3.124	>100	12.5	25
14	50.0	>50	3.124	>100	6	25
18	50.0	>50	3.124	>100	12.5	25
20	50.0	>50	3.124	>100	12.5	25
22	50.0	>50	3.124	>100	6	25
25	50.0	>50	3.124	>100	12.5	25
26	50.0	>50	3.124	>100	12.5	25
29	50.0	>50	3.124	>100	12.5	25
30	25.0	>50	3.124	>100	12.5	25
31	50.0	>50	3.124	>100	12.5	25
32 (2010)	50.0	>50	3.124	>100	12.5	25
32 (2015)	50.0	>50	3.124	>100	3	25
34	50.0	>50	3.124	>100	3	25
35	50.0	>50	3.124	>100	12.5	25
42	50.0	>50	3.124	>100	3	25
43	50.0	>50	3.124	>100	3	25
45	50.0	>50	3.124	>100	6	25
47	25.0	>50	3.124	>100	12.5	25
50	50.0	>50	1.562	>100	12.5	25
51	50.0	>50	1.562	>100	6	25
54	50.0	>50	3.124	>100	12.5	25
56 (2004)	12.5	>50	3.124	>100	12.5	25
56 (2013)	50.0	>50	3.124	>100	12.5	25
62	50.0	>50	3.124	>100	6	25
64	50.0	>50	3.124	>100	12.5	25
64 (2004)	25.0	>50	3.124	>100	12.5	25
65	50.0	>50	3.124	>100	6	25
66	50.0	>50	3.124	>100	12.5	25

	EAST (mg/mL)		EVVP (mg/mL)		EP (mg/mL)	
**Isolate**	**MIC**	**MBC**	**MIC**	**MBC**	**MIC**	**MBC**
67	25.0	>50	3.124	>100	12.5	25
71	50.0	>50	1.562	>100	12.5	25
77	25.0	>50	3.124	>100	12.5	25

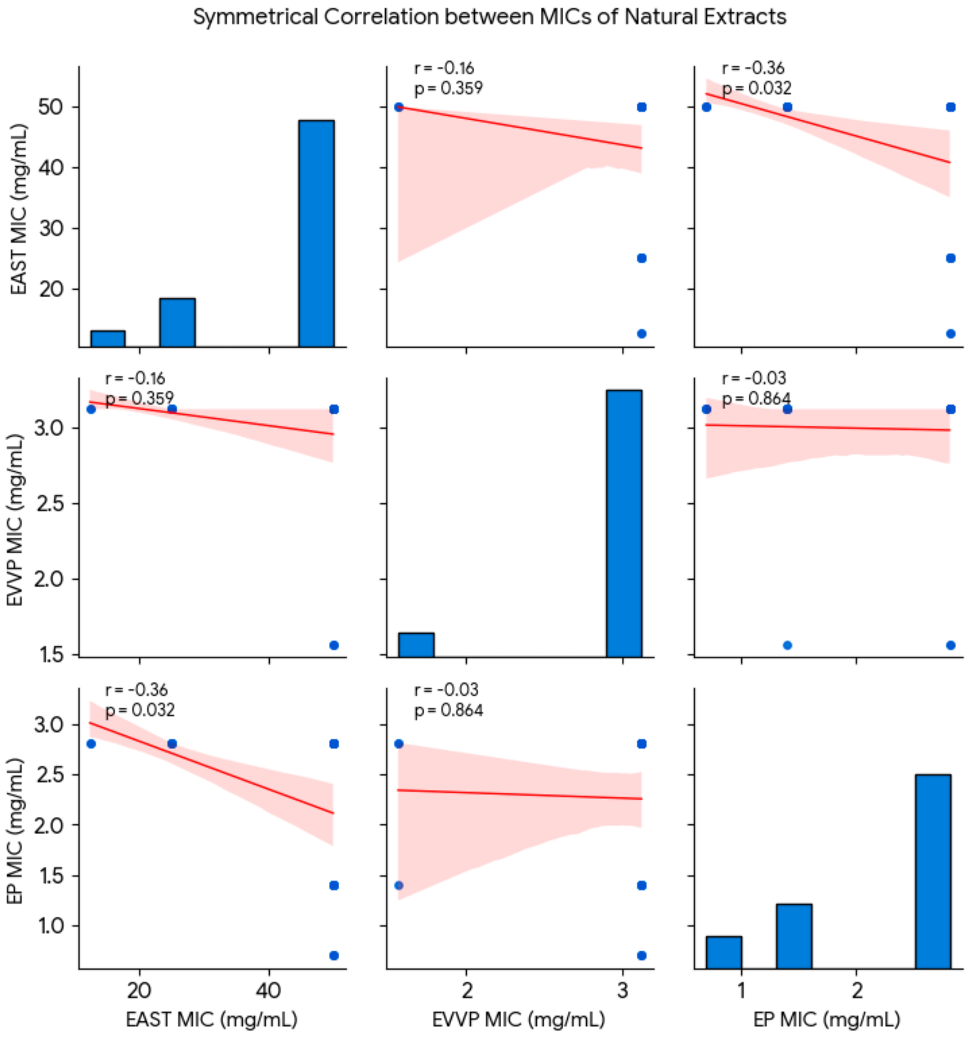

Supplementary Figure S1. Pairwise correlation analysis of Minimum Inhibitory Concentrations (MICs) for the three natural extracts. The off-diagonal panels show the relationships between the MICs of each pair of extracts (garlic-EAST, grape-EVVP, and propolis-EP). Histograms on the diagonal show the distribution of MIC values for each individual extract against the 36 MRSA isolates. Each scatter plot includes a linear regression fit (red line) and is annotated with the Pearson correlation coefficient (r) and the corresponding p-value.
